# The evolutionally-conserved function of group B1 Sox family members confers the unique role of Sox2 in mouse ES cells

**DOI:** 10.1186/s12862-016-0755-4

**Published:** 2016-08-31

**Authors:** Hitoshi Niwa, Akira Nakamura, Makoto Urata, Maki Shirae-Kurabayashi, Shigehiro Kuraku, Steven Russell, Satoshi Ohtsuka

**Affiliations:** 1 Laboratory for Pluripotent Stem Cell Studies, RIKEN Center for Developmental Biology (CDB), 2-2-3 Minatojima-minamimachi, Chuo-ku, Kobe 650-0047 Japan; 2 Department of Pluripotent Stem Cell Biology, Institute of Molecular Embryology and Genetics, Kumamoto University, 2-2-1 Honjo, Chuo-ku, Kumamoto 860-0811 Japan; 3 Department of Germline Development, Institute of Molecular Embryology and Genetics, Kumamoto University, 2-2-1 Honjo, Chuo-ku, Kumamoto 860-0811 Japan; 4 Noto Marine Laboratory, Institute of Nature and Environmental Technology, Kanazawa University, Ogi 34-11, Noto-cho, Hohsu, Ishikawa, 927-0553 Japan; 5 Sugashima Marine Biological Laboratory, Graduate School of Science, Nagoya University, Sugashima-cho 429-63, Toba C, Mie 517-0004 Japan; 6 Phyloinformatics Unit, RIKEN Center for Life Science Technologies (CLST), 2-2-3 Minatojima-minamimachi, Chuo-ku, Kobe 650-0047 Japan; 7 Department of Genetics, University of Cambridge, Downing Street, Cambridge, CB2 3EH UK; 8 Present address: Department of Life Science, Medical Research Institute, Kanazawa Medical University, 1-1 Daigaku, Uchinada, kahoku, Ishikawa, 920-0293 Japan

**Keywords:** Pluripotent stem cells, Sox2, Evolution, Co-option

## Abstract

**Background:**

In mouse ES cells, the function of Sox2 is essential for the maintenance of pluripotency. Since the Sox-family of transcription factors are well conserved in the animal kingdom, addressing the evolutionary origin of *Sox2* function in pluripotent stem cells is intriguing from the perspective of understanding the origin of pluripotency.

**Results:**

Here we approach this question using a functional complementation assay in inducible *Sox2*-null ES cells. Assaying mouse Sox proteins from different Groups, we found that only Group B1 and Group G proteins were able to support pluripotency. Interestingly, invertebrate homologs of mammalian Group B1 Sox proteins were able to replace the pluripotency-associated function of mouse *Sox2*. Moreover, the mouse ES cells rescued by the *Drosophila SoxNeuro* protein are able to contribute to chimeric embryos.

**Conclusions:**

These data indicate that the function of mouse *Sox2* supporting pluripotency is based on an evolutionally conserved activity of the Group B1 Sox family. Since pluripotent stem cell population in developmental process could be regarded as the evolutional novelty in vertebrates, it could be regarded as a co-optional use of their evolutionally conserved function.

**Electronic supplementary material:**

The online version of this article (doi:10.1186/s12862-016-0755-4) contains supplementary material, which is available to authorized users.

## Background

Pluripotency is a unique feature of the cells found in early vertebrate embryos. Pluripotent stem cells give rise to all cell types of the organism, including germ cells, but, unlike zygotes, they do not have the ability to give rise organisms autonomously [1]. The pluripotent phenotype is primarily determined by the expression of a set of pluripotency-associated transcription factors, as demonstrated by the induction of pluripotency in somatic cells transfected with four transcription factors to give rise to induced pluripotent stem (iPS) cells [2]. Of these four transcription factors, Oct3/4 (encoded by *Pou5f1*) and Sox2 are known to be essential for maintaining pluripotency in mouse embryonic stem (ES) cells [3, 4]. In contrast, the functions of Klf4 and Myc are dispensable for pluripotency, but primarily support self-renewal in the absence of the cytokine leukemia inhibitory factor (LIF) [5–8].

Pluripotent stem cell populations have been definitively identified in mammalian embryos, but their presence in other vertebrate embryos remains unclear, with no pluripotent stem cell lines yet isolated from other taxa. Frog animal cap cells behave similarly to pluripotent cells, but have never been shown to yield stem cells capable of propagating in vitro [9]. The absence of pluripotent stem cells is evident in ascidian embryos, since blastomeres exhibit mosaic behavior upon artificial separation [10]. Likewise there is no evidence of pluripotent stem cells in well studied invertebrates, including fly and nematode. It therefore seems that the presence of a pluripotent stem cell population in the early embryo is a novelty exclusive to higher vertebrates.

Addressing the evolutionary origins of transcription factor functions coupled with pluripotency is an interesting challenge, since it may provide insights into the origins of the pluripotency-associated transcription factor network. Oct3/4 belongs to the POU family and its evolutionary history has been addressed in several studies [11–13]. The POU family of transcription factors are categorized into 6 classes and *Oct3/4* (*Pou5f1*) is a member of class V. Although homologs of the other classes of POU family members can be found in the genomes of invertebrates, such as *Caenorabditis elegans* and *Drosophila melanogaster*, there is no class V POU family member in any invertebrate genome studied to date, strongly suggesting that *Oct3/4* is a genetic novelty in the vertebrate genome. In fish genomes, *Pou2 (Pou5f3)* is the evolutionarily oldest member of the class V POU family, but is not syntenic with *Oct3/4* in mammals, and the functional complementation assays using fish *Pou2* in mouse ES cells revealed only a weak ability to substitute for the function of *Oct3/4* in supporting pluripotency. In the case of amphibian, there are three class V POU family members in the genome of *Xenopus tropicalis* and these are found in tandem at a region syntenic with the *Pou2* locus in the fish genome. One of these, *Xlpou91*, is known to rescue *Oct3/4* function, providing a signature of molecular evolution. Monotreme *Oct3/4* is a true ortholog that shares conserved synteny with other mammalian *Oct3/4* genes, and functions to replace native Oct3/4 in mouse ES cells, although the homology of its POU domain at the amino acid level in comparison to the mouse ortholog is not markedly different from its homology to the POU domain of zebrafish *Pou2*. Interestingly, the monotreme genome also possesses the *Pou2* ortholog with conserved synteny, indicating that *Oct3/4* and *Pou2* are paralogous. Recently, it was suggested that a lizard genome may include an ortholog of *Oct3/4* in a conserved syntenic position, but to date no functional analysis has been reported. The above observations indicate that *Oct3/4* is a relatively recent evolutionary acquisition whose ancestor evolved in vertebrates as *Pou2*, and subsequently underwent a duplication to generate *Oct3/4* as a new class V POU family member.

Sox2 belongs to the Sry-related high mobility group (HMG)-box (Sox) family of transcription factors, whose members are characterized by a conserved HMG box DNA binding domain related to the mammalian testis determining factor Sry. Members of the Sox family bind to consensus DNA sequences and act as either transcriptional activators or repressors [14]. In the mouse genome, there are 20 members of the Sox family categorized into 8 groups. Sox2 belongs to Group B1, which has two other members, Sox1 and Sox3. The Group B1 Sox family is well conserved and has been identified in virtually all multi-cellular vertebrate and invertebrate animals, where they share conserved functions in neural development. In pluripotent stem cells, it is known that Sox2 interacts with Oct3/4 to activate the transcription of target genes [15, 16]. When and how Sox2 acquired its function as a partner of Oct3/4 is an interesting but as yet unanswered question, although it has been suggested that this function is unique to Group B1 Sox family members. Interestingly, it has been shown that in *Drosophila* the Group B protein Dichaete interacts with the Class III POU protein Vvl during neural development and in this role the mouse Sox2 gene is able to functionally substitute for the fly protein [17], suggesting that Sox-POU interactions are ancient.

In the present study, we sought to reveal the molecular basis of the function of Sox2 in pluripotent stem cells and shed light on its evolutionary origins. We employed a functional complementation assay in mouse ES cells in which endogenous *Sox2* alleles were disrupted by gene targeting, while a tetracycline-regulatable *Sox2* transgene supports pluripotency [4]. We tested mouse Sox family members from different groups along with Group B1 Sox family members from various organisms for their ability to substitute for mouse *Sox2* in maintaining pluripotency. We find that a single evolutionarily-conserved amino acid is important for the function of SoxB1 proteins in maintaining mouse ES cell self-renewal. Finally, we report that the *Drosophila* SoxNeuro protein can substitute for the functions of mouse Sox2 required to maintain pluripotency.

## Results

### *Sox1*, *Sox3*, and *Sox15* can replace the function of *Sox2* in mouse ES cells

**Fig. 1:**
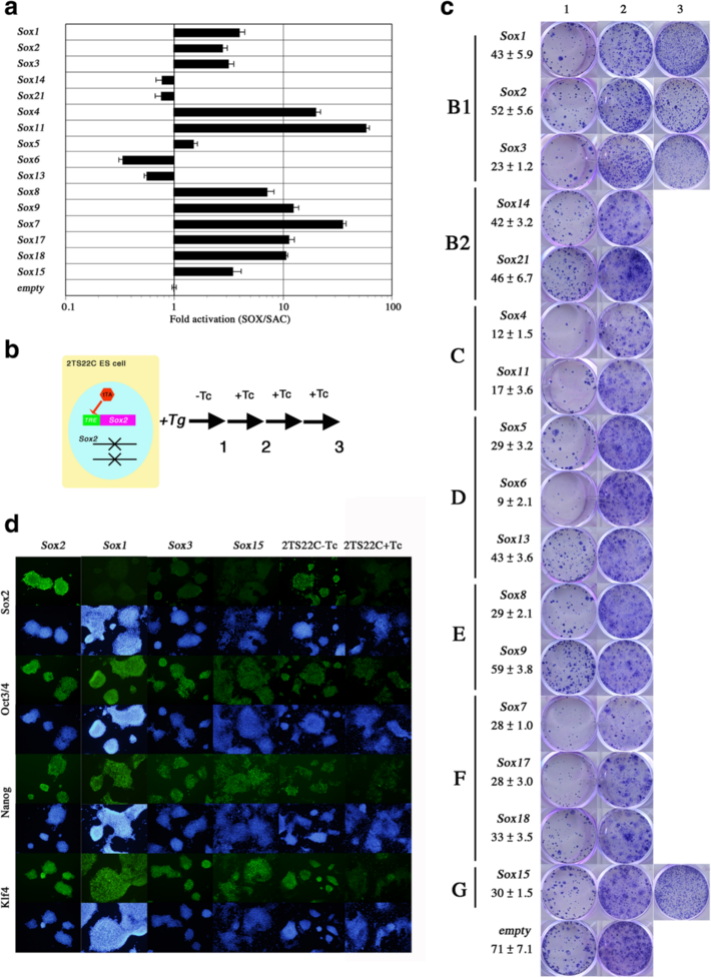
Sox2 rescue ability of mouse Sox factors. **a** Reporter gene assays of mouse Sox factors. The ability to activate or repress the promoter activity via consensus SOX binding motifs was assayed and the relative activities are indicated as ‘fold activation’ on the log scale. A value under 1.0 indicates that the factor functions as a transcriptional repressor. Each assay represents the mean of 3 replicates, error bars mean standard deviations. **b** Cartoon of Sox2 rescue experiments. 2TS22C ES cells were transfected with an expression vector containing a mouse Sox factor followed by culture without tetracycline (Tc) in FCS-medium to give a pool of transfectants without Sox2. Transfectants were serially passaged in KSR-medium with Tc at a density 3x10^3^/well, and evaluated for their ability to form stem cell colonies. Numbers 1–3 indicate the time points of the evaluation demonstrated in Columns 1–3 of C. **c** Sox2 rescue ability. The numbers of primary colonies and standard deviation obtained with each *Sox* gene are indicated under the gene symbol. Column1 shows colonies of primary transfectants grown without Tc. Column 2 shows secondary colonies grown in the presence of Tc. Stem cell colonies were recognized their tightly packed morpohology. Column 3 shows colonies at passage 3 in the presence of Tc if they yielded stem cell colonies at this stage. Stem cell colony formation at this stage indicates rescue of *Sox2*-null ES cells. **d** Immunostaining of rescued ES cells. Rescued ES cells at passage 4 in the presence of Tc were stained for Sox2, Oct3/4, Nanog and Klf4. The absence of Sox2 staining with rabbit anti-Sox2 polyclonal antibody in *Sox1*, *Sox3* and *Sox15* transfectants confirmed rescue. 2TS22C ES cells cultured with or without Tc for 4 days are shown as positive and negative controls, respectively

The mouse genome contains 20 Sox family members, which are divided into eight groups based on the amino acid sequence of the conserved HMG-box [18] (Additional file 1: Figure S1A). We selected one to three members from each group except group A, which contains *Sry* as its sole member. The full set of open reading frames encoding the Sox family members were isolated from mouse cDNA pools or FANTOM cDNA clones, and subcloned into the PiggyBac (PB) expression vector *pPBCAG-cHA-IB* (see Materials and Methods). Their functions were first tested by reporter assays in HeLa cells. Either the recognition site shared by Sox family members (AACAAAG: SOX) or a mutated version (CCGCGGT: SAC) were placed as 7 tandemly arranged copies upstream of the Herpes Simplex Virus *thymidine kinase* (HSV-*tk*) minimal promoter driving expression of a *luciferase* reporter gene. We separately co-transfected HeLa cells with one of these reporters along with a Sox gene expression vector and determined the ratio of SOX to SAC reporter activities in the presence of 16 different Sox proteins. From these assays we confirmed that Sox6, Sox13, Sox14, and Sox21 act as transcriptional repressors, since they repress the expression of the Sox reporter. As previously reported, the remaining Sox factors function as activators [14, 19] (Fig. 1a), confirming the validity of our Sox expression vectors.

The Sox expression vectors were then introduced into 2TS22C mouse ES cells, these lack endogenous *Sox2* and are maintained by transgenic *Sox2* expression regulated by the tet-OFF system [4]. These ES cells maintain self-renewal in the absence of tetracycline (Tc) but cease self-renewal and undergo differentiation to trophectoderm upon addition of Tc. We introduced the Sox expression vectors using the PiggyBac system, that results in efficient integration of transgenes into the mouse genome [20], and individual lines were cultured with or without Tc. If an introduced Sox family member is capable of replacing the function of *Sox2*, the cells will be able to continue self-renewal over several passages in the presence of Tc (Fig. 1b). In line with this expectation, we found that expression vectors containing *Sox1, Sox3, Sox4, Sox5, Sox8, Sox9, Sox11, Sox13, Sox14, Sox15, Sox18, and Sox21* yielded comparable numbers of stem cell colonies after culturing for seven days in the absence of Tc (Fig. 1c). In contrast, both *Sox6* and *Sox7* evidently produced differentiated cell colonies (Additional file 1: Figure S1B). We found that *Sox6* expression resulted in many trophectoderm-like colonies, perhaps attributable to its strong function as a transcriptional repressor (Fig. 1a), which could compete with the function of *Sox2*. In contrast, *Sox7* induced parietal endoderm-like cells, in line with our recent report [21]. Stable cell lines carrying each Sox factor were dissociated and 1 × 10^4^ cells were seeded in the presence of Tc, allowing selective propagation of the rescued stem cells. At the first passage, most of the Sox vector containing cells showed obviously differentiated phenotypes as did the cells containing the empty vector control, whereas *Sox2* transfectants maintained an undifferentiated phenotype. At this period, only *Sox1* and *Sox3* supported stem cell colony formation as efficiently as *Sox2*. After the third passage in this condition, the rescuing ability of each Sox factor was evaluated by stem cell colony formation. Among the 15 Sox factors tested, only three (*Sox1, Sox3* and *Sox15*) showed the ability to restore ES cell self-renewal although *Sox15* supported very few stem cell colonies at the first passage (Fig. 1c).

Absence of Sox2 in these rescued ES cells was confirmed by immunostaining with anti-Sox2 antibody (Fig. 1d) and the expression of Sox1, Sox3 or Sox15 from the transgenes were confirmed by immunostaining with the relevant specific Sox antibody (Additional file 1: Figure S1C-E). We found that the rescued cells maintained expression of the pluripotency-associated transcription factors Oct3/4, Nanog and Klf4 (Fig. 1d), indicating they behave as self-renewing ES cells. These data indicate that the function of Sox2 in supporting mouse ES cell self-renewal is highly specific and shared with few other Sox family proteins.

### A single conserved amino acid in the HMG box is responsible for the unique function of Sox2

**Fig. 2:**
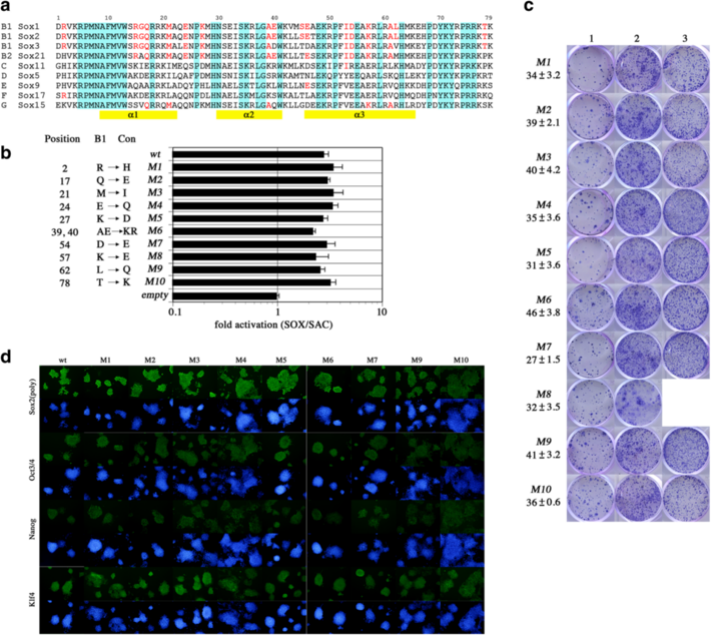
Rescue with mutated mouse Sox2 proteins. **a** Amino acid alignments of HMG-box from mouse Sox family members. Amino acids conserved in all Sox factors are marked with blue, and amino acids unique to SoxB1 are highlighted in red. **b** Reporter gene assays of mutant proteins. The position and identity of amino acid substitutions in each line are shown on the left: all proteins show comparable ability to activate the Sox2 reporter. Each assay represents the mean of 3 replicates, error bars mean standard deviations. **c** Rescue ability of altered Sox2 proteins. The numbers of primary colonies and standard deviation obtained with each mutant Sox2 protein are indicated under the ID. Colums represent: 1 – primary transfectants in the absence of Tc. 2 – Secondary colonies in the presence of Tc. 3 – Colonies at passage 3 in the presence of Tc. Only the M8 (K57E) mutant failed to provide wild-type Sox2 function. **d** Immunostaining of rescued ES cells. Rescued ES cells at passage 4 in the presence of Tc were stained for Sox2, Oct3/4, Nanog and Klf4. Mutant forms of Sox2 were detected by a goat polyclonal anti-Sox2 antibody

Sox1 and Sox3 belong to Group B1 and show high amino acid identity with Sox2 in the HMG box. Sox15 belongs to Group G and it shows much lower sequence similarity to the Sox2 HMG box than the Sox14 and Sox21 repressors that cannot replace Sox2 function. We note that Group G proteins are phylogenetically closest to Group B [14, 19] (Additional file 1: Figure S1A), suggesting that unique amino acid(s) conserved among Groups B1 and G may confer their unique rescue function. The HMG box consists of 79 amino acids that form three α-helix structures mediating binding to the minor groove of target DNA sequences [19]. Many amino acids are conserved across all Sox family members, distinguishing them from other HMG-box-containing transcription factors (Additional file 1: Figure S1A). Sequence alignments also reveal amino acids shared by the members of particular groups that are not found in other groups. Group B1 Sox proteins share 14 unique amino acids that are rarely found in other groups: these are good candidates for conferring the unique ES cell functions. To test the contribution of these amino acids to the Sox2 ES cell functions, we made a series of 10 mouse Sox2 mutant proteins in which each unique amino acid was substituted for an amino acid shared by other Sox groups (Fig. 2a): nine of the mutants changed a single amino acid and one changed two. All the mutant proteins showed comparable ability to activate the Sox-dependent reporter in the HeLa cell assay, confirming that they retain the wild type ability to bind the target sequence and to activate transcription (Fig. 2a). When tested in 2TS22C ES cells, nine of the 10 mutants were able to provide wild-type *Sox2* function and maintain self-renewal (Fig. 2b). In contrast, the one mutant protein that failed to rescue self-renewal carried a lysine to glutamate substitution at position 57 in the third helix of the HMG domain. Sox15, the Group G protein that rescues Sox2 self-renewal activity, also contains a lysine at position 57 (Additional file 1: Figure S1A). Interestingly, this amino acid was previously reported to confer the differential activity of Sox2 and Sox17 in reprogramming somatic cells to iPS cells [22]. As reported, we confirmed that replacement of E57 with K in Sox17 conferred the ability to support self-renewal of *Sox2*-null ES cells (Additional file 2: Figure S2A-C). Together, these data indicate that a unique amino acid shared by Group B1 and Group G proteins primarily confers the self-renewal function of Sox family genes in ES cells.

### Self-renewal in mouse ES cells is evolutionarily conserved in Group B1 Sox proteins

**Fig. 3:**
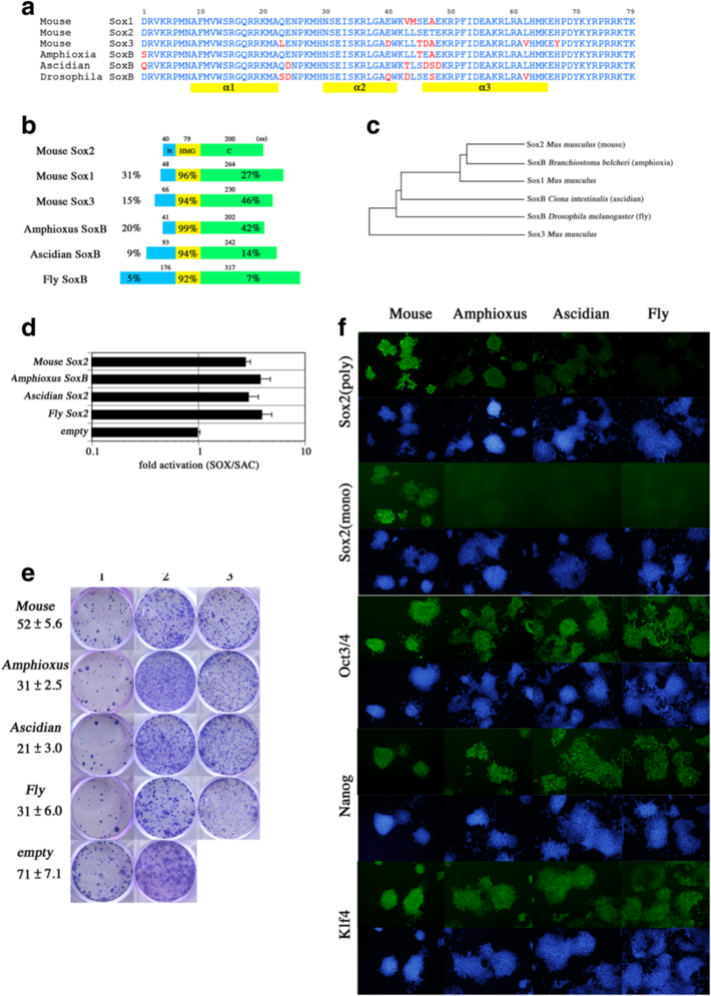
Rescue ability of invertebrate SoxB homologs. **a** Amino acid alignments of the HMG-box of mouse GroupB1 family members and SoxB homologs from amphioxus, ascidian (Ci-SoxB1) and fluitfly (SoxN). Non-homologous amino acids are highlighted in red, the 3 helices of the DNA binding domain are indicated as yellow bars. **b** Cartoon of SoxB protein structures. All share a very similar 79 amino acid HMG-box but the N- and C-terminal regions vary in their length and degree of homology to Sox2 (shown as %). **c** A maximum likelihood phylogenetic tree of SoxB homologs. **d** Reporter gene assays with SoxB homologs. Relative activities are indicated as ‘fold activation’ on the log scale. Each assay represents the mean of 3 replicates, error bars mean standard deviations. All factors show comparable ability to activate the Sox2 reporter. **e** ES cell rescue assays. The numbers of primary colonies and standard deviation obtained with each GroupB Sox protein are indicated under the ID Colums represent: 1 – primary transfectants in the absence of Tc. 2 – Secondary colonies in the presence of Tc. 3 – Colonies at passage 3 in the presence of Tc. All Sox factors assayed showed rescue ability. **f** Immunostaining of the rescued ES cells. Sox2 (poly) indicates staining with goat anti-Sox2 antibody which shows weak cross-reactivity with SoxB homologs, except for the fly SoxN protein. Sox2 (mono) indicates staining with a mouse anti-Sox2 monoclonal antibody specifically reactive with mouse Sox2, confirming the proper loss of mouse Sox2 in the rescued ES cells

Next we tested the functions of SoxB1 homologs isolated from various animals. The vertebrate Sox2 orthologs found at syntenic genomic locations share a highly conserved amino acid sequence along the entire molecule and retain the ability to replace the function of Sox2 in mouse ES cells (data not shown). We tested invertebrate SoxB1 homologs for their ability to replace the function of mouse Sox2 in mouse ES cells. We selected SoxB1 proteins that showed the highest homology to the mouse Sox2 HMG box from the genomes of amphioxus (*Branchiostoma japonicum*), an ascidian (*Ciona intestinalis type A;* renamed to *Ciona robusta* by Brunetti et al. [23]) and the fruitfly (*Drosophila melanogaster*) (Fig. 3a). The ascidian and fluitfly SoxB homologs we chose were annotated as *Ci-SoxB1* and *SoxNeuro* (*SoxN*), respectively. These SoxB1 homologs share conservation only within the HMG box, and show no significant sequence homology in other coding regions (Fig. 3b). A phylogenetic analysis with amino acid sequences from the HMG domains confirmed these proteins were correctly related (Fig. 3c). We isolated the sequence for the SoxB1 homologs from their genomes by PCR and subcloned the coding sequence into the PiggyBac expression vector. All three of these SoxB1 homologs showed the ability to activate the Sox reporter in HeLa cells at levels comparable to mouse *Sox2* (Fig. 3d).

We then assessed the ability of the three Group B1 proteins to functionally substitute for mouse *Sox2* in ES cells. We performed the rescue assay using 2TC22C ES cells described above and found that amphioxus *SoxB1,* ascidian *Ci-SoxB1* and fly *SoxN* possess the ability to support self-renewal of *Sox2*-null ES cells (Fig. 3e). The immunostaining analysis of these ES cells confirmed the maintenance of Oct3/4, Nanog and Klf4 expression of with loss of endogenous mouse Sox2 (Fig. 3f). Therefore, the evolutionally-conserved function of the SoxB1 homologs is sufficient to maintain mouse ES cells self-renewal.

### Fly SoxN maintains pluripotency of mouse ES cells

**Fig. 4:**
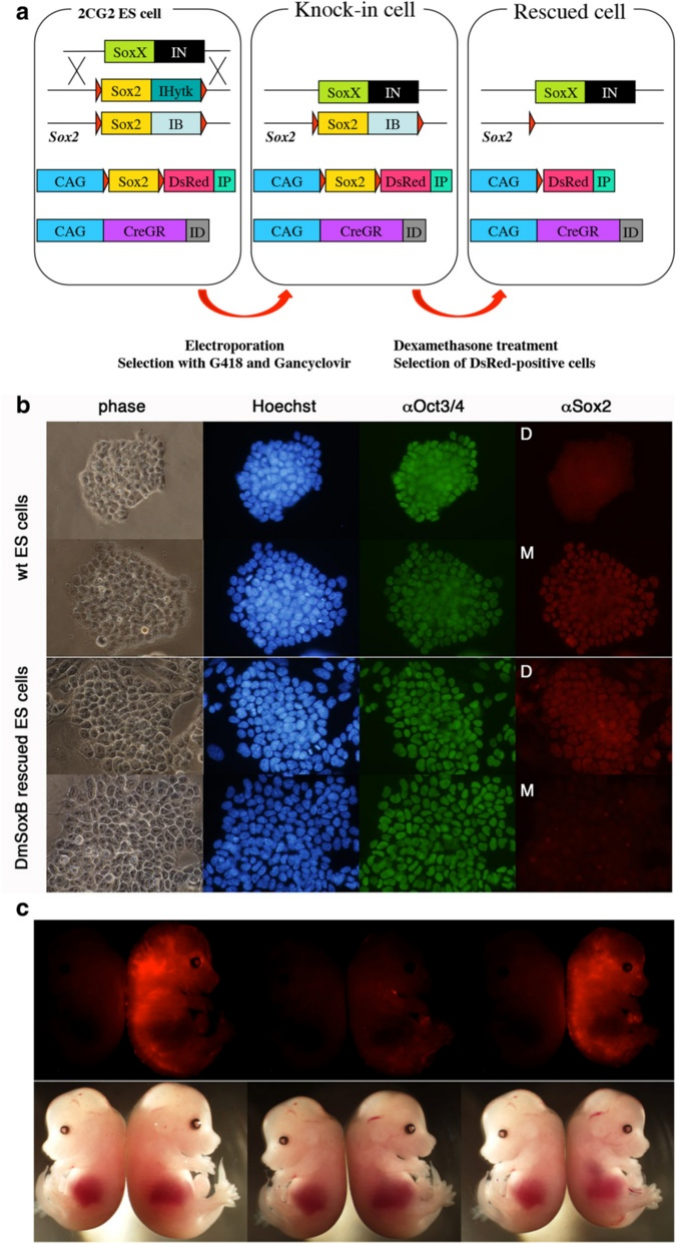
Mouse ES cells maintained by *Drosophila* SoxN retain pluripotency. **a** A strategy to eatablish mouse ES cells maintained by *Drosophila* SoxN. 2CG2 ES cells were engineered according to the steps shown. The resulting ES cells, designated Hae ES cells, express *Drosophila* SoxN under the regulation of the endogenous Sox2 promoter. **b** Immunostaining of Hae ES cells. In the column of αSox2, D and M indicate the staining with anti-SoxN antisera and mouse anti-Sox2 monoclonal antibody, respectively. Hae ES cells express *Drosophila* SoxN but not mouse Sox2. Nuclear signals indicate the antibody staining whereas the cytoplasmic fluorescence signals were derived from DsRed. **c** Chimeric embryos generated by injection of Hae ES cells expressing DsRed (*right side* of each panel) with the negative control (*left side*)

These data indicate that, at least in culture, invertebrate SoxB1 factors possess the ability to support self-renewal of mouse ES cells. To confirm the ability of the invertebrate SoxB1 to support pluripotency of mouse ES cells *in vivo* we utilised an alternative strategy. 2CG2 ES cells carry *loxP* sites in both endogenous *Sox2* alleles as well as the expression vectors of the floxed *Sox2*, dexamethasone-inducible *Cre* recombinase (*CreGR*) and *DsRed* under the regulation of the constitutively-active *CAG* promoter [4] (Fig. 4a). 2CG2-derived ES cells have a confirmed ability to contribute to chimeric embryos and we therefore used them as a host for further engineering to generate mouse *Sox2*-null ES cells rescued by *Drosophila SoxN*. We first replaced one of the endogenous floxed *Sox2* alleles by *Drosophila SoxN* without the *loxP* site using homologous recombination. Then these ES cells were cultured in the presence of dexamethasone to activate the Cre recombinase activity for deletion of the remained floxed *Sox2* allele and the floxed *Sox2* transgene. The correct removal of these floxed regions were confirmed by the PCR analysis of genomic DNA and by the constitutive expression of the *DsRed* transgene (data not shown). As the result, we obtained mouse ES cells that lack the expression of mouse *Sox2* but express *Drosophila SoxN* under the control of endogenous mouse *Sox2* regulatory sequences.

These ES cells, designated as Hae ES cells, grow normally and maintain the expression of Oct3/4 (Fig. 4b). We confirmed the presence of SoxN and absence of Sox2 by immunostaining with specific antibodies (Fig. 4b). When we injected these cells into blastocysts followed by uterine transfer to pseudo-pregnant females, we obtained chimeric embryos with obvious systemic distribution of DsRed-positive cells (Fig. 4c). These results indicate that Hae ES cells retain pluripotency and are able to contribute to many lineages in the developing mouse embryo.

## Discussion

Tissue-specific transcription factors act as the primary determinants of cell phenotypes in multi-cellular organisms. Genetic evidence from model organisms suggests that multiple transcription factors cooperate through direct and indirect interactions to determine a single phenotype. Several evolutionary novelties arose during the evolution of animals to generate the complex cellular architectures that characterize these organisms. Such events were presumably coupled with the evolution of new transcription factor functions. The presence of a pluripotent stem cell population during early developmental stages is a unique feature of higher vertebrates that facilitates flexible developmental processes. We and others previously showed that the *Pou5f1* gene encoding Oct3/4, one of the factors essential for conferring the pluripotent phenotype, may have co-evolved with the acquisition of a pluripotent cell population during vertebrate evolution [11–13]. In contrast, in the case of its partner Sox2, not only do all vertebrates possess Sox2 orthologs, but SoxB1 genes sharing high sequence homology in the HMG box DNA binding domain are found across all metazoan genomes, indicating an very early origin during animal evolution [24]. In the present study we show that invertebrate SoxB1 homologs possess the ability to functionally substitute for Sox2 and support the self-renewing state in mouse ES cells, indicating that the unique function of Sox2 in pluripotent stem cells is based on a conserved function of metazoan SoxB1 proteins.

We identified a single amino acid, K57 positioned in the third α-helix of the DNA binding domain, is responsible for conferring the unique function of Sox2 in supporting pluripotency, raising questions about the role of this amino acid. Interestingly, a previous report indicated that this amino acid is positioned in the interaction surface with Oct3/4 in ternary Sox-Oct complex formed on the *Utf1* enhancer [25]. That study also showed that two amino acid substitutions, including K57E, abolished the interaction with Oct3/4, which agrees well with our finding that mutated Sox2 proteins carrying the K57E substitution were no longer able to maintain pluripotency. Interestingly, this unique amino acid is conserved in all group B Sox family members, in mouse Group G proteins and in the invertebrate SoxB proteins we assayed. The evidence that Sox15 is able to replace the function of Sox2 supports the importance of this amino acid. Moreover, as reported previously, the introduction of this amino acid to Sox17 conferrs the ability to functionally substitute for Sox2 in iPS cell assays [22], supporting the view that this amino acid, at least in part, is able to confer unique pluripotency functions of Sox2. Clearly K57 is not a sufficient for Sox2-specific pluripotency functions in ES cells: for example, the Group B2 proteins Sox14 and Sox21 also possess this amino acid but do not support self-renewal of *Sox2*-null ES cells. However, this is not surprising since these two proteins are known transcriptional repressors [26], which we confirmed in our HeLa cell reporter assay. In contrast, all three invertebrate Group B1 proteins we tested act as transcriptional activators in the same assay. These data suggested that the HMG-box with K57 and a transactivation domain are minimal requirements for Sox2 activity supporting pluripotency. We previously reported the failure of hydra SoxB to replace the function of Sox2, which could be due to the lack of proper transactivation domain that can act in mouse ES cells since it also possess K57 [27].

The functional conservation of SoxB proteins in supporting ES cell self-renewal and pluripotency is remarkable but not unique, with several reports of conserved function between mouse and fly now published. In a famous example, expression of the mouse *Pax6* gene can induce the formation of ectopic eye-like structures, mimicking the phenotypes elicited by the fly homolog *eyeless* [28]. The mutant phenotype of *Drosophila tinman* was partially rescued by the transgenic expression of a mouse homologue *Nkx2.5* [29]. In the case of Sox2, we have shown that mouse Sox2 transgenes can rescue aspects of neural development in *Dichaete* null mutants [17]. However, in all these cases the functional conservation is manifest in homologous developmental processes: *Pax6* and *eyeless* normally function during eye development, *Nkx2.5* and *tinman* participate in heart development, and both *Sox2* and *Dichaete* have well documented roles in neural development. In contrast, here we show that *Drosophila SoxN* can replace *Sox2* function in pluripotent stem cells, for which there is no homologous cells or tissues in *Drosophila*. To our knowledge a comparable case has only been reported with the mouse GroupE Sox gene *Sox10* and its role in neural crest development. In this case the mouse gene was replaced by the *Drosophila* Group E gene *Sox100B* and, as we find here, although the fly lacks any conterpart to the vertebrate neural crest, the fly gene is able to provide substantial function in the absence of *Sox10* [30]. We believe these cases provide evidence that supports the idea that conserved functions of homologous genes can be integrated into new functions acquired during evolution that generate biological novelty.

How was the conserved Sox2 function integrated into the pluripotency-associated transcription factor network? Previous work showed that the expression of *Sox2* in ES cells is supported by the ES-specific enhancers. SRR1 and SRR2, located at the 5′ and 3′ proximal regions of *Sox2*, were first identified [31]. SRR1/N-2 is regulated by Oct3/4 [32] and SRR2 is activated by Oct3/4 and Sox2 in ES cells [31]. Comparative genome analysis of human, mouse and chicken *Sox2* orthologs revealed several conserved elements that possess this enhancer activity [33]. Of these, SRR2 is conserved in chicken but not in Xenopus and zebrafish, suggesting its evolutionally new origin [33]. In addition, the recent reports demonstrated that the distal super-enhancer element possesses higher contribution to the transcriptional activation of Sox2 in ES cells although the evolutional origin of this element has not been characterized [34, 35]. We consider that the acquisition of novel regulatory elements would be necessary since Sox2 has evolutionarily conserved functions essential for neural development that restrict the flexibility to acquire new functions by modifying the protein sequence. Interestingly, Sox2 function is also important for self-renewal of mouse trophoblast stem (TS) cells. We previously reported that Sox2 has an alternative partner, Tfap2c rather than Oct3/4, and regulates different sets of target genes in ES and TS cells [36]. Since the trophectoderm lineage gives rise to the placenta, which is an obvious evolutional novelty acquired by eutherians, it will be of interest to dissect the function of Sox2 in mouse TS cells using a approaches similar to those described here. Such an analysis should provide further insights into the molecular mechanisms used during evolution to generate new functions for tissue-specific transcription factors and regulatory networks without increasing the number of genes in the genome.

## Conclusion

We demonstrate that the function of Sox2 to maintain self-renewal of mouse ES cells is specifically shared by Group B1 of Sox-family members. The invertebrate SoxB1 members also possess this activity although they lack pluripotent stem cell population in developmental process, indicating that the function of SoxB1 factor in pluripotent stem cells is a co-optional use of the evolutionally-conserved function.

## Methods

### Vectors

All ORFs for mouse Sox family members and Group B Sox factors from other species were isolated by PCR with KOD-Fx (Toyobo) or Pfx polymerase (Invitrogen) using primer pairs with either mouse ES cDNA, FANTOM clones or genomic DNA as listed in Additional file 3: Table S1. Amplicons were subcloned into the PiggyBac expression vector *pPBCAG-cHA-IB*, which was made by insertion of *CAG-IB* into the PiggyBac transposon unit [20]. Mutagenesis of mouse *Sox2* and other Sox family members was performed by PCR with KOD-Fx with the primer pairs listed in Additional file 4: Table S2. The ORFs of all of vectors were sequenced and confirmed free of unexpected mutations.

### ES cell culture and complementation assay

2TS22C ES cells were cultured in GMEM supplemented with 10 % Knockout Serum Replacement (KSR; Invitrogen), 1 % fetal calf serum (FCS), 1 × non-essential amino acids (Nakarai), 1 mM Sodium pyruvate (Nakarai), 10^−4^ M 2-mercaptoethanol and 10^3^ U/ml of mouse LIF on gelatin coated surface. For the complementation assay, 3 × 10^4^ 2TS22C ES cells were seeded in wells of 48 well plates. The following day, cells were transfected with 1 μg of the PiggyBac Sox expression vector and 1 μg of pCAGGS-PBase [20] using Lipofectoamine 2000 (Invitrogen), and replated into four wells of a 12-well plate. Cells were selected with 10 μg/ml of Blasticidin S (Invivogen) from one to seven days after transfection. Three wells of cells were stained with Leischman stain to count the numbers of stem cell colonies. One well of cells was dissociated and 3 × 10^3^ cells seeded into a well of a 12-well plate either in the presence or absence of 1 μg/ml of tetracycline. After six days, one-fifth of the dissociated cells were replated into a well of a 12-well plate followed by culture with tetracycline for 6 days. Stem cell colonies were scored by Leischman staining as well as re-seeding into either 12-well or 48-well plates for RNA preparation and immunostaining, respectively.

### Reporter assay

HeLa cells were cultured in GMEM with 10 % FCS. 10^4^ HeLa cells were seeded into a well of a 96-well plate. The following day, three wells of cells were transfected with 0.5 μg of the PiggyBac Sox expression vector, 0.5 μg of either SOX (AACAAAG) × 7 (tandem repeat)-tk-luc or SAC (CCGCGGT) × 7 (tandem repeat)-tk-luc and 10 ng of pRL-SV (Promega) followed by the culture for 24 h. The cells were then tested for luciferase activity using the Dual luciferase assay kit (Promega) with Centro LB 960 luminometer (Berthold).

### Immunostaining

Cells were fixed with 4 % paraformaldehyde in PBS for 30 min at 4°C, followed by permealization with 0.2 % TritonX100 in PBS for 10 min at RT. These cells were incubated with the following primary antibodies overnight at 4°C; mouse monoclonal anti-Sox2 (R&D Systems, MAB2018), 1:1000; goat polyclonal anti-Sox2 (Neuromics, GT15098), 1:1000; mouse monoclonal anti-Oct3/4 (Santa Cruz, 5279), 1:1000; rat monoclonal anti-Nanog (e-Bioscience, MLC-51), 1:1000; rabbit polyclonal anti-Klf4 (Santa Cruz, 20691), 1:300; anti-SoxN antisera (Ferrero et al., 2014), 1:300; rabbit polyclonal anti-Sox1 (Chemicon, ab5768), 1:300; rabbit polyclonal anti-Sox3 (Santa Cruz 20089), 1:300; goat polyclonal anti-Sox15 (Santa Cruz, 17354), 1:300: goat polyclonal anti-Sox17 (R&D Systems, AF1924), 1:300. After washing, cells were incubated with appropriate secondary antibodies conjugated with Alexa-488 for 1 h at RT with Hoechst 33258 and fluorescent images were taken on an Olympus OX-71 equipped with a CCD camera.

### Establishment of Hae ES cells

The *Drosophila SoxN* knock-in vector was generated by replacing *loxP*-mouse *Sox2* ORF-*IRES-Bsd-pA-loxP* in a previously described knockout vector [4] with *Drosophila SoxN* ORF-*IRES-neo-pA*. The linealized plasmid DNA of this knock-in vector was transfected into 2CG2 ES cells by electroporation followed by selection with G418 and Gancyclovir. Surviving clones were screened by genomic DNA-PCR to identify knock-in cell lines. Sucessful knock-in ES cells were cultured with dexamethasone for activation of CreGR followed by the replating at clonal density with G418 and puromycin. The DeRed-positive clones were isolated and viability in culture with blasticidin S was tested. Blasticidin S-sensitive clones were screened by genomic DNA-PCR to obtain *Sox2*-null ES cell lines maintained by *SoxN*, we designate these as Hae ES cells.

### Production of chimeric embryos

Dissociated Hae ES cells were introduced into a C57BL6 blastocyst by microinjection, which was then transferred to the uterus of a pseudopregnant female ICR mouse. Embryos were collected at 13.5 dpc and the contribution of the injected ES cells to the chimera evaluated by fluorescence microscopy. All animal experiments conformed to our Guidelines for the Care and Use of Laboratory animals and were approved by the Institutional Committee for Laboratory Animal Experimentation (RIKEN Kobe Institute).

## Additional files

Additional file 1:
**Figure S1.** Analysis of *Sox2*-null ES cells rescued by wild-type Sox factors. **A** Morphology of primary transfectants with *Sox6* and *Sox7*. Transfection of *Sox6* expression vectors caused differentiation of large flat trophectoderm-like cells, whereas with *Sox7* spindle cells resembling parietal endoderm were occasionally observed. **B** Immunostaining of *Sox2*-null ES cells rescued by *Sox1* with anti-Sox1. Ectopic expression of *Sox1* in rescued ES cells was confirmed. 2TS22C ES cells cultured without Tc serve as a negative control. **C** Immunostaining of *Sox2*-null ES cells rescued by *Sox3* with anti-Sox3. Ectopic expression of *Sox3* in rescued ES cells was confirmed. 2TS22C ES cells cultured without Tc serve as a negative control. **D** Immunostaining of *Sox2*-null ES cells rescued by *Sox15* with anti-Sox15. Ectopic expression of *Sox15* in rescued ES cells was confirmed. 2TS22C ES cells cultured without Tc serve as a negative control. (PDF 2664 kb) Additional file 2:
**Figure S2.** Analysis of *Sox2*-null ES cells rescued by Sox 17 carrying the E57K mutation. **A** Rescue assay of Sox17E57K in which E57 was replaced by K. The numbers of the primary colonies of transfectants with the standard deviation are indicated under the gene symbols. Column1 shows colonies of primary transfectants grown without Tc. Column 2 shows secondary colonies grown in the presence of Tc. Stem cell colonies were recognized their tightly packed morpohology. Column 3 shows colonies at passage 3 in the presence of Tc if they yielded stem cell colonies at this stage. **B** Immunostaining of rescued ES cells. The rescued ES cells at passage 4 in the presence of Tc were stained for Sox2, Oct3/4, Nanog and Klf4. The absence of Sox2 staining with goat anti-Sox2 polyclonal antibody in *Sox17E57K* transfectants confirmed the rescue. **C** Immunostaining of *Sox2*-null ES cells rescued by *Sox17E57K* with anti-Sox17 antibody*.* Ectopic expression of *Sox17E57K* in rescued ES cells was confirmed. 2TS22C ES cells cultured without Tc serve as a negative control (PDF 932 kb) Additional file 3:
**Table S1.** PCR primers for isolation of Sox genes. (PDF 42 kb) Additional file 4:
**Table S2.** PCR primers for mutagenesis of mouse Sox genes. (PDF 35 kb)

## References

[1] Niwa H (2007). How is pluripotency determined and maintained?. Development.

[2] Takahashi K, Yamanaka S (2006). Induction of pluripotent stem cells from mouse embryonic and adult fibroblast cultures by defined factors. Cell.

[3] Niwa H, Miyazaki J, Smith AG (2000). Quantitative expression of Oct-3/4 defines differentiation, dedifferentiation or self-renewal of ES cells. Nat Genet.

[4] Masui S, Nakatake Y, Toyooka Y, Shimosato D, Yagi R, Takahashi K, Okochi H, Okuda A, Matoba R, Sharov AA (2007). Pluripotency governed by Sox2 via regulation of Oct3/4 expression in mouse embryonic stem cells. Nat Cell Biol.

[5] Cartwright P, McLean C, Sheppard A, Rivett D, Jones K, Dalton S (2005). LIF/STAT3 controls ES cell self-renewal and pluripotency by a Myc-dependent mechanism. Development.

[6] Jiang J, Chan YS, Loh YH, Cai J, Tong GQ, Lim CA, Robson P, Zhong S, Ng HH (2008). A core Klf circuitry regulates self-renewal of embryonic stem cells. Nat Cell Biol.

[7] Niwa H, Ogawa K, Shimosato D, Adachi K (2009). A parallel circuit of LIF signalling pathways maintains pluripotency of mouse ES cells. Nature.

[8] Smith KN, Singh AM, Dalton S (2010). Myc represses primitive endoderm differentiation in pluripotent stem cells. Cell Stem Cell.

[9] Okabayashi K, Asashima M (2003). Tissue generation from amphibian animal caps. Curr Opin Genet Dev.

[10] Venuti JM, Jeffery WR (1989). Cell lineage and determination of cell fate in ascidian embryos. Int J Dev Biol.

[11] Niwa H, Sekita Y, Tsend-Ayush E, Grutzner F (2008). Platypus Pou5f1 reveals the first steps in the evolution of trophectoderm differentiation and pluripotency in mammals. Evol Dev.

[12] Frankenberg S, Pask A, Renfree MB (2010). The evolution of class V POU domain transcription factors in vertebrates and their characterisation in a marsupial. Dev Biol.

[13] Frankenberg S, Renfree MB (2013). On the origin of POU5F1. BMC Biol.

[14] Kamachi Y, Kondoh H (2013). Sox proteins: regulators of cell fate specification and differentiation. Development.

[15] Sharov AA, Masui S, Sharova LV, Piao Y, Aiba K, Matoba R, Xin L, Niwa H, Ko MS (2008). Identification of Pou5f1, Sox2, and Nanog downstream target genes with statistical confidence by applying a novel algorithm to time course microarray and genome-wide chromatin immunoprecipitation data. BMC Genomics.

[16] Yuan H, Corbi N, Basilico C, Dailey L (1995). Developmental-specific activity of the FGF-4 enhancer requires the synergistic action of Sox2 and Oct-3. Genes Dev.

[17] Soriano NS, Russell S (1998). The Drosophila SOX-domain protein Dichaete is required for the development of the central nervous system midline. Development.

[18] Schepers GE, Teasdale RD, Koopman P (2002). Twenty pairs of sox: extent, homology, and nomenclature of the mouse and human sox transcription factor gene families. Dev Cell.

[19] Bowles J, Schepers G, Koopman P (2000). Phylogeny of the SOX family of developmental transcription factors based on sequence and structural indicators. Dev Biol.

[20] Guo G, Yang J, Nichols J, Hall JS, Eyres I, Mansfield W, Smith A (2009). Klf4 reverts developmentally programmed restriction of ground state pluripotency. Development.

[21] Kinoshita M, Shimosato D, Yamane M, Niwa H (2015). Sox7 is dispensable for primitive endoderm differentiation from mouse ES cells. BMC Dev Biol.

[22] Jauch R, Aksoy I, Hutchins AP, Ng CK, Tian XF, Chen J, Palasingam P, Robson P, Stanton LW, Kolatkar PR (2011). Conversion of Sox17 into a pluripotency reprogramming factor by reengineering its association with Oct4 on DNA. Stem Cells.

[23] Brunetti R, Gissi C, Pennati R, Caicci F, Gasparini F, Manni L (2015). Morphological evidence that the molecularly determined Ciona intestinalis type A and type B are different species: Ciona robusta and Ciona intestinalis. J Zool Syst Evol Res.

[24] Phochanukul N, Russell S (2010). No backbone but lots of Sox: Invertebrate Sox genes. Int J Biochem Cell Biol.

[25] Remenyi A, Lins K, Nissen LJ, Reinbold R, Scholer HR, Wilmanns M (2003). Crystal structure of a POU/HMG/DNA ternary complex suggests differential assembly of Oct4 and Sox2 on two enhancers. Genes Dev.

[26] Uchikawa M, Kamachi Y, Kondoh H (1999). Two distinct subgroups of Group B Sox genes for transcriptional activators and repressors: their expression during embryonic organogenesis of the chicken. Mech Dev.

[27] Hemmrich G, Khalturin K, Boehm AM, Puchert M, Anton-Erxleben F, Wittlieb J, Klostermeier UC, Rosenstiel P, Oberg HH, Domazet-Loso T (2012). Molecular signatures of the three stem cell lineages in hydra and the emergence of stem cell function at the base of multicellularity. Mol Biol Evol.

[28] Gehring WJ (1996). The master control gene for morphogenesis and evolution of the eye. Genes Cells.

[29] Ranganayakulu G, Elliott DA, Harvey RP, Olson EN (1998). Divergent roles for NK-2 class homeobox genes in cardiogenesis in flies and mice. Development.

[30] Cossais F, Sock E, Hornig J, Schreiner S, Kellerer S, Bosl MR, Russell S, Wegner M (2010). Replacement of mouse Sox10 by the Drosophila ortholog Sox100B provides evidence for co-option of SoxE proteins into vertebrate-specific gene-regulatory networks through altered expression. Dev Biol.

[31] Tomioka M, Nishimoto M, Miyagi S, Katayanagi T, Fukui N, Niwa H, Muramatsu M, Okuda A (2002). Identification of Sox-2 regulatory region which is under the control of Oct-3/4-Sox-2 complex. Nucleic Acids Res.

[32] Uchikawa M, Ishida Y, Takemoto T, Kamachi Y, Kondoh H (2003). Functional analysis of chicken Sox2 enhancers highlights an array of diverse regulatory elements that are conserved in mammals. Dev Cell.

[33] Iwafuchi M, Yoshida Y, Onichtchouk D (2011). M leichsenring, W Driever, T Takemoto, M Uchikawa, Y Kamachi, H Kondoh: The Pou5f1/Pou3f-dependent but SoxB-independent regulation of conserved enhancer N2 initiates Sox2 expression during epiblast to neural plate stages in vertebrates. Dev Biol.

[34] Li Y, Rivera CM, Ishii H, Jin F, Selvaraj S, Lee AY, Dixon JR, Ren B (2014). CRISPE reveals a distal super-enhancer required for Sox2 expression in mouse embryonic stem cells. PLoS One.

[35] Zhou HY, Katsman Y, Dhaliwal NK, Davidson S, Macpherson NN, Sakthidevi M, Collura F, Mitchell JA (2014). A Sox2 distal enhancer cluster regulates emrbyonic stem cell differentiation potential. Genes Dev.

[36] Adachi K, Nikaido I, Ohta H, Ohtsuka S, Ura H, Kadota M, Wakayama T, Ueda HR, Niwa H (2012). Context-dependent wiring of Sox2 regulatory networks for self-renewal of embryonic and trophoblast stem cells. Mol Cell.

