# Supplementary figures and images for: The evolutionally-conserved function of group B1 Sox family members confers the unique role of Sox2 in mouse ES cells
Source: BMC Evol Biol. 2016 Aug 31;16(1):173. doi: 10.1186/s12862-016-0755-4 (PMC5007870; doi:10.1186/s12862-016-0755-4)

**A**

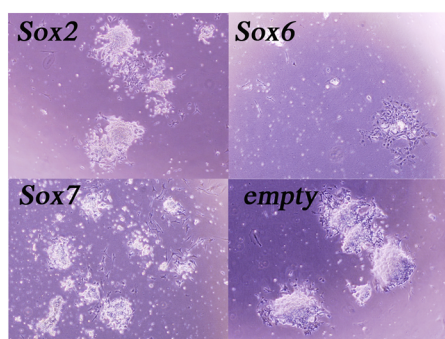

**B**

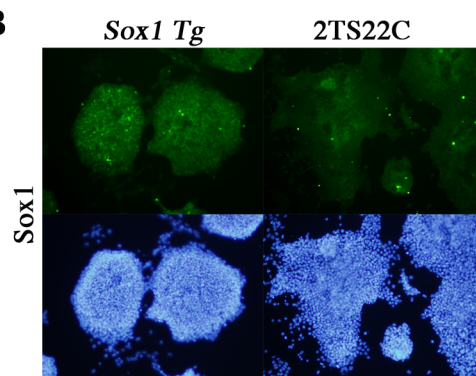

**C**

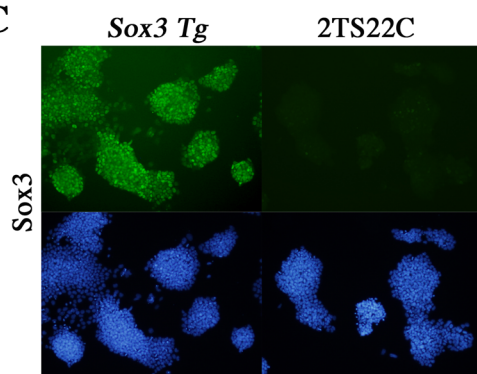

**D**

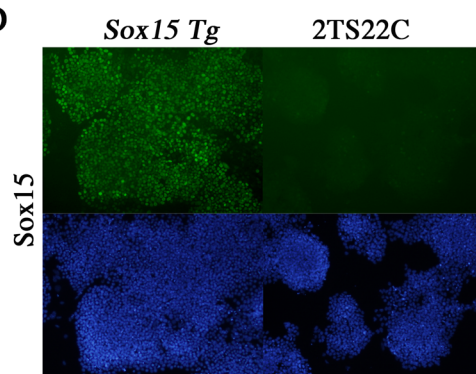

Supplement: Additional file 1: — Figure S1. Analysis of Sox2-null ES cells rescued by wild-type Sox factors. A Morphology of primary transfectants with Sox6 and Sox7. Transfection of Sox6 expression vectors caused differentiation of large flat trophectoderm-like cells, whereas with Sox7 spindle cells resembling parietal endoderm were occasionally observed. B Immunostaining of Sox2-null ES cells rescued by Sox1 with anti-Sox1. Ectopic expression of Sox1 in rescued ES cells was confirmed. 2TS22C ES cells cultured without Tc serve as a negative control. C Immunostaining of Sox2-null ES cells rescued by Sox3 with anti-Sox3. Ectopic expression of Sox3 in rescued ES cells was confirmed. 2TS22C ES cells cultured without Tc serve as a negative control. D Immunostaining of Sox2-null ES cells rescued by Sox15 with anti-Sox15. Ectopic expression of Sox15 in rescued ES cells was confirmed. 2TS22C ES cells cultured without Tc serve as a negative control. (PDF 2664 kb) [file 12862_2016_755_MOESM1_ESM.pdf]

**A**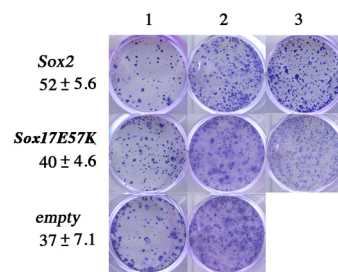**B**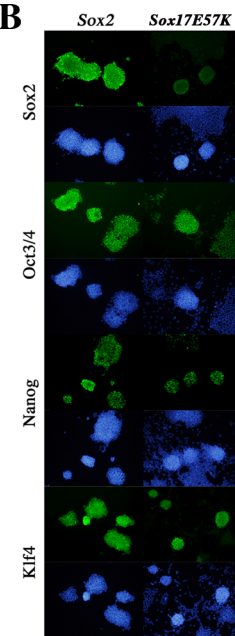**C**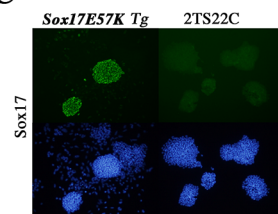

Supplement: Additional file 2: — Figure S2. Analysis of Sox2-null ES cells rescued by Sox 17 carrying the E57K mutation. A Rescue assay of Sox17E57K in which E57 was replaced by K. The numbers of the primary colonies of transfectants with the standard deviation are indicated under the gene symbols. Column1 shows colonies of primary transfectants grown without Tc. Column 2 shows secondary colonies grown in the presence of Tc. Stem cell colonies were recognized their tightly packed morpohology. Column 3 shows colonies at passage 3 in the presence of Tc if they yielded stem cell colonies at this stage. B Immunostaining of rescued ES cells. The rescued ES cells at passage 4 in the presence of Tc were stained for Sox2, Oct3/4, Nanog and Klf4. The absence of Sox2 staining with goat anti-Sox2 polyclonal antibody in Sox17E57K transfectants confirmed the rescue. C Immunostaining of Sox2-null ES cells rescued by Sox17E57K with anti-Sox17 antibody. Ectopic expression of Sox17E57K in rescued ES cells was confirmed. 2TS22C ES cells cultured without Tc serve as a negative control (PDF 932 kb) [file 12862_2016_755_MOESM2_ESM.pdf]
