# Supplementary material for: The evolutionally-conserved function of group B1 Sox family members confers the unique role of Sox2 in mouse ES cells
Source: BMC Evol Biol. 2016 Aug 31;16(1):173. doi: 10.1186/s12862-016-0755-4 (PMC5007870; doi:10.1186/s12862-016-0755-4)
Supplement: Additional file 3: — Table S1. PCR primers for isolation of Sox genes. (PDF 42 kb) [file 12862_2016_755_MOESM3_ESM.pdf]

Table 1. PCR primers for isolation of Sox genes.

Gene		Sequence
Amphioxus SoxB	F	ATCTCGAGCCACCATGATGGAGACAGCCGTGCACCCGGCCCAG
	R	ATGCGGCCGCTCACATGTGACTGAGGGGAACTGTACC
Ascidian SoxB	F	AACTTCTCCGTCCCTACTCCACC
	R	ACGAACTCCTACATATGCGCAG
Fly SoxNeuro	F	AATGTCGACCACCATGCTGACCATGGAATCGGATATGAAAG
	R	ATGCGGCCGCTCACATGTGTGCCAGCGGCGCCATCGCC
Mouse Sox1	F	ACTGCAGCCGCCCAGATGTACAGCATGATG
	R	ACTGCAGCCGCCCAGATGTACAGCATGATG
Mouse Sox3	F	AAGTCGACATAACTTCGTATAG
	R	GCACTAGTTTTAGATGTGGGTCAGCGG
Mouse Sox4	F	ATCTCGAGCCACCATGGTACAACAGACC
	R	GCACTAGTTCAGTAGGTGAAGACCAGG
Mouse Sox5	F	AAAGTCGACTTTTCCAGCATGCTTACTGAC
	R	AAGCGGCCGCCCTTATCAGTTGGCTTGTCC
Mouse Sox6	F	CCTCGAGTAACTAAGAATGTCTTCCAAGCAAG
	R	CCGCGGCCGCTCCTCAGTTGGCACTGACAGGCTC
Mouse Sox7	F	CTCGAGCCACCATGGCCTCGCTGCTG
	R	GCACTAGTTTTATGACACACTGTAGCTG
Mouse Sox8	F	AAAGTCGACGCGTGGCCCCGATGCTGGACA
	R	AAGCGGCCGCCTCAGGGTCGGGTCAGGGTG
Mouse Sox9	F	AAAGTCGACGGGCTCGCGTATGAATCTCCT
	R	AAGCGGCCGCTTTTCTCTTCTCAGGGTCTG
Mouse Sox11	F	ATCTCGAGCCACCATGGTGCAGCAGG
	R	GAGCGGCCGCTTCAATACGTGAACACCAG
Mouse Sox13	F	CCTCGAGCGCTAGCACCATGGTGAACTGCAC
	R	CCGCGGCCGCGGGATCAGTCTGTGAGTACCACCAG
Mouse Sox14	F	AAAGTCGACGCCGGGACCATGTCCAAACCT
	R	AAGCGGCCGCTTGGGGTTACATGGCCGTAG
Mouse Sox15	F	AAAGTCGACTCCAACCCATGGCGCTGACCA

	R	AAGCGGCCGCCAGTTAAAGGTGGGTTACTG
Mouse Sox17	F	GTCTCGAGAGCCATGAGCAGCCCGGATG
	R	GCACTAGTTTTAAATGTCGGGGTAGTTG
Mouse Sox18	F	AAAGTCGACCAGCTGGAATGCAGAGATCGC
	R	AAGCGGCCGCAAGGACAGTGTCTAGCCTGA
Mouse Sox21	F	CCTCGAGGCGACAGCATGTCCAAGCCTGTGG
	R	AACTAGTGGGGTCATAGCGCGGCAGCGTAGGC

F: forward primer, R: reverse primer
