# Supplementary material for: The evolutionally-conserved function of group B1 Sox family members confers the unique role of Sox2 in mouse ES cells
Source: BMC Evol Biol. 2016 Aug 31;16(1):173. doi: 10.1186/s12862-016-0755-4 (PMC5007870; doi:10.1186/s12862-016-0755-4)
Supplement: Additional file 4: — Table S2. PCR primers for mutagenesis of mouse Sox genes. (PDF 35 kb) [file 12862_2016_755_MOESM4_ESM.pdf]

Table 2. PCR primers for mutagenesis of mouse Sox genes.

Gene		Sequence
Sox2M1	F	CGTCAAGAGGCCCATGAACGCCTTC
	R	TGGTCCGGGCTGTTCTTCTGGTTG
Sox2M2	F	GGGGGGAGCGGCGTAAGATGGCCCAG
	R	GGGACCATACCATGAAGGCGTTCATG
Sox2M3	F	GGGGGCAGCGGCGTAAGATAGCCCAGGAG
	R	GGGACCATACCATGAAGGCGTTCATG
Sox2M4	F	CATAACTCGGAGATCAGCAAGCGCCTG
	R	CATCTTGGGGTTCTGCTGGGCCATCT
Sox2M5	F	CATAACTCGGAGATCAGCAAGCGCCTG
	R	CATGTCGGGGTTCTCCTGGGCCATCT
Sox2M6	F	GCTGGAAACTTTTGTCCGAGACCGAGA
	R	GCTTGCCCAGGCGCTTGCTGATCTCCGAG
Sox2M7	F	GCGCTCTGCACATGAAGGAGCACCC
	R	GCAGCCGCTTGGCCTCTTCGATGAACGGC
Sox2M8	F	GCGCTCTGCACATGAAGGAGCACCC
	R	GCAGCCGCTCGGCCTCGTCGATGAACGGC
Sox2M9	F	GCGCTCAGCACATGAAGGAGCACCC
	R	GCAGCCGCTTGGCCTCGTCGATGAACGGC
Sox2M10	F	GGCGGAAAAAAAAGACGCTCATGAAGAAG
	R	GCGGCCGGTATTTATAATCCGGGTG
Sox17M8R	F	CGTGCAGCATATGCAGGACCACCC
	R	CGTAGCCGCTTGGCCTCTTCCACGAAGGG

F: forward primer, R: reverse primer
